# A Singular Plug in the External Meatus

**Published:** 1879-10

**Authors:** F. C. Hotz

**Affiliations:** 181 Clark St.


### Article VIII.

### *A Singular Plug in the External Meatus.*

On the 14th of August a gentleman connected with a pantomime troupe, consulted me in regard to an increasing deafness and tinnitus which he first noticed about six weeks previously. He always had good hearing ; was never troubled by ear-ache, as far as he could remember. His rôle is that of the clown, who has to appear upon the stage all white from head to foot. Before each performance he had to paint his face with a whitening made of bismuth and pure lard, which, after the performance, was rubbed off again with lard. The auricles, of course, had to undergo the same process; and it seems that at every cleaning off of the auricle, a little of the bismuth and lard dropped into the external meatus, where, in the course of years, it accumulated to large deposits. For, upon examination, I found each meatus filled to the external orifice with a greyish-yellow substance, which, in consistence and cohesive power, resembled exactly half-dry putty. Injections of warm water made no marked impression on it until it had been treated with a solution of bicarbonate of soda. As far as the alkaline lotion, instilled repeatedly during the day, could penetrate within 24 hours, the plug was soft enough on the next day to be easily broken up and removed by injections. In three successive sittings both meatus were cleaned; the hearing was fully restored, and the tinnitus had disappeared. The removed mass was carefully collected in a glass; it was a mixture of cerumen, lard, fine hairs and a heavy, yellow-white powder, which, in water, sank to the bottom of the glass, and thus could be easily separated from the greasy matter. This powder, weight 1.65 grams, was recognized as oxychloride of bismuth by chemical tests.

F. C. Hotz, m.d.

181 Clark St.

---

O. C. Oliver, m.d., of Chicago, has been appointed Assistant Physician of the Elgin Hospital for Insane. A better selection could not have been made.

Prof. Chas. T. Parkes, m.d., who has been absent in Europe for five months, engaged in study, returned with Prof. Byford.

Two Legs.—According to the *Lyon Médical*, a *cause célèbre* is on trial before the "Supreme Court of Colombia," [sic.] in the United States, where a man, who lost two legs in the late war, is suing to recover them from the general government.

